# The health workforce crisis in Bangladesh: shortage, inappropriate skill-mix and inequitable distribution

**DOI:** 10.1186/1478-4491-9-3

**Published:** 2011-01-22

**Authors:** Syed Masud Ahmed, Md Awlad Hossain, Ahmed Mushtaque RajaChowdhury, Abbas Uddin Bhuiya

**Affiliations:** 1 Research and Evaluation Division, BRAC, 75 Mohakhali, Dhaka-1212, Bangladesh; 2 James P Grant School of Public Health, BRAC University, 66 Mohakhali, Dhaka-1212, Bangladesh; 3 ICDDRB, Mohakhali, Dhaka-1212, Bangladesh

## Abstract

**Background:**

Bangladesh is identified as one of the countries with severe health worker shortages. However, there is a lack of comprehensive data on human resources for health (HRH) in the formal and informal sectors in Bangladesh. This data is essential for developing an HRH policy and plan to meet the changing health needs of the population. This paper attempts to fill in this knowledge gap by using data from a nationally representative sample survey conducted in 2007.

**Methods:**

The study population in this survey comprised all types of currently active health care providers (HCPs) in the formal and informal sectors. The survey used 60 unions/wards from both rural and urban areas (with a comparable average population of approximately 25 000) which were proportionally allocated based on a 'Probability Proportion to Size' sampling technique for the six divisions and distribution areas. A simple free listing was done to make an inventory of the practicing HCPs in each of the sampled areas and cross-checking with community was done for confirmation and to avoid duplication. This exercise yielded the required list of different HCPs by union/ward.

**Results:**

HCP density was measured per 10 000 population. There were approximately five physicians and two nurses per 10 000, the ratio of nurse to physician being only 0.4. Substantial variation among different divisions was found, with gross imbalance in distribution favouring the urban areas. There were around 12 unqualified village doctors and 11 salespeople at drug retail outlets per 10 000, the latter being uniformly spread across the country. Also, there were twice as many community health workers (CHWs) from the non-governmental sector than the government sector and an overwhelming number of traditional birth attendants. The village doctors (predominantly males) and the CHWs (predominantly females) were mainly concentrated in the rural areas, while the paraprofessionals were concentrated in the urban areas. Other data revealed the number of faith/traditional healers, homeopaths (qualified and non-qualified) and basic care providers.

**Conclusions:**

Bangladesh is suffering from a severe HRH crisis--in terms of a shortage of qualified providers, an inappropriate skills-mix and inequity in distribution--which requires immediate attention from policy makers.

## Background

Human resource for health (HRH) is the critical limiting factor determining the health of the population besides socioeconomic, behavioural and environmental factors [1,2]. Globally, there is a close correlation between the concentration of qualified health workers (doctors, nurses, dentists and midwives together) and key health outcomes such as immunization coverage, primary health care outreach, and infant, under-5 and maternal survival. This is because "in health systems, workers function as gatekeepers and navigators for the effective, or wasteful application of all other resources such as drugs, vaccines and supplies" [3]. The shortage of qualified health workers, especially in low-income countries, has drawn attention in recent times, as it seriously threatens the attainment of the millennium development goals (MDGs) [4,5].

The countries of WHO's South-East Asia Region also face several common health workforce related problems and issues concerning shortage, skill-mix, migration, work environment, knowledge-base and other areas amply articulated in the 'Dhaka Declaration' [6]. Bangladesh is no exception in this regard and it is one of the countries with 'severe shortages' of health workers [3]. Given the shortage of supply of qualified health care providers in Bangladesh, patients, especially the poor and the disadvantaged, mostly seek health care from the nonqualified providers in the informal sector [7,8]. On the demand side, due to lack of health awareness, the overall health service consumption (from any source) in Bangladesh is low compared to other developing countries, as is level of need [9,10]. Evidence shows that overall levels of per capita consumption of essential service package (ESP) would have to increase by 40% in order to achieve the higher average level of other developing countries [11].

To develop an effective, efficient and equitable health system for meeting the goal of improved and equitable population health, human resources for health (HRH) should be appropriate in relation to number, skill-mix, and distribution with optimum competency and motivation. There is a lack of comprehensive, nationally representative data on HRH in the formal and informal sectors in Bangladesh. This is essential for developing an HRH policy and plan and its implementation to meet the changing health needs of the population. A population-based, nationally representative survey covering all types of health care providers in the formal and informal sectors was done in 2007 by Bangladesh Health Watch (BHW) to fill in this knowledge gap [12]. BHW is a civil society initiative "to regularly and systematically measure and monitor the country's progress and performance in health". This paper presents data from this survey and discusses its implications for HRH problems in Bangladesh.

## Materials and methods

### Study population and sampling

The study population in this survey comprised of all types of health care providers (HCPs)--allopathic and non-allopathic, trained and untrained, and in public or private sector--who were currently active in providing healthcare services to the community in the study areas. The survey used 60 primary sampling units (PSUs, a cluster of around 200 households) drawn randomly from the nationally representative 1000 PSUs that are used by the Bangladesh Bureau of Statistics (BBS) for its Sample Vital Registration System, yielding estimates up to the level of district [13]. The number of sample PSUs (n = 60) was conveniently determined given constraints in time and resources. From the total number of PSUs in each of the division, the required number of sample PSUs was taken randomly, following a PPS (Probability Proportion to Size) sampling technique. Table 1 shows this proportional allocation of the sample PSUs by division. Thus, the sampling provided representative estimates of the density of health care providers for the country as a whole, for the urban and the rural areas separately, and for each of the six administrative divisions (note that the study was done prior to the 2010 creation of a seventh Bangladeshi division). Since a PSU may not be large enough to have sufficient HCPs in terms of number as well as diversity, we used the Union and the Ward (lowest administrative units having comparable population size of around 25 000) containing the selected PSU as the sampling unit for the rural and the urban areas respectively. Data collection was done during July-September 2007.

**Table 1:** Number of sample Primary Service Units (PSUs) by division and rural/urban areas according to PPS (Probability Proportion to Size)

Division	Total PSUs enumerated by Bangladesh Bureau of Statistics for Sample Registration Survey		Number of sample PSUs taken		
	**All**	**Urban**	**All**	**Rural**	**Urban**

Barisal	79	11	4.7 ≈ 5	4	1
Chittagong	172	41	10.3 ≈ 10	8	2
Dhaka	307	105	18.4 ≈ 18	12	6
Khulna	125	25	7.5 ≈ 8	6	2
Rajshahi	263	39	15.8 ≈ 16	14	2
Sylhet	55	7	3.3 ≈ 3	2	1
Rural	774	---	46.5 ≈ 46	---	---
Urban	225	---	13.5 ≈ 14	---	---

Total	1000		60	46	14

Note: Urban area included the statistical metropolitan area (SMA), municipality area and other urban areas (e.g. 'thana sadar' area which has the quality of municipality).

### Inventory of health care providers

All the villages, markets and health facilities/centres under each PSU (Union/Ward) were visited by the field enumerators (social science graduates) who were recruited and trained by the research team. They started by identifying the initial batch of key informants through informal discussion (asking questions such as "Who in your locality can give valid information about the number and types of the HCPs?") with community members in the markets and villages. Further key informants were then identified from this information using a 'snowball' technique. The key informants were then asked to list all the practicing HCPs they knew in the locality (free listing), and an inventory of practicing HCPs was made for each of the geographical areas visited. The key-informants sometimes provided information about the HCPs' names in different ways (e.g., nick name, family name, title, etc.). These were cross-checked with other key informants and village people for proper identification, and to avoid duplication and omission, especially in the case of the informal sector providers. Also, they visited the residences of the HCPs for on-the-spot confirmation whenever confusion arose. It was relatively easy to get the list of working HCPs from the administrative authorities in different public and private sector healthcare facilities. The enumerators also frequently checked authenticity of information with the HCPs whenever feasible. During this process, they explained the purpose of the inventory and sought their cooperation for improving the validity of the data. Finally, this exercise yielded a list of different HCPs by Union/Ward (PSU).

### Categorization of the informal health care providers

The informal health care providers (not registered with any government regulatory body) were categorized into the following groups:

1) Semi-qualified allopathic providers: include providers who have received training of varying duration from a formal institution in the public or private sector such as the non-profit NGOs.

a. Para-professionals: comprised of the medical assistants who completed a three-year medical assistant training programme from a public institution, mid-wives (family welfare visitor (FWV)) with 18 months training in midwifery and clinical contraception management from public/private institutions, and lab-technicians/physiotherapists

b. Community health workers (CHWs) from both public and non-governmental organisation (NGO) sectors. The CHWs in the NGO sector outnumber those in the public sector by a ratio of 2:1 [12]. CHWs have variable lengths of basic preventive and curative health care training, from various health care providing NGOs mainly, but also from the public sector.

2) Unqualified allopathic providers: included in this category are village doctors and drug store sales people/drug vendors.

a. The village doctors (also known as rural medical practitioner, RMP) mostly received short training (from a few weeks to a few months) on some common illnesses/conditions, from semi-formal private institutions which are unregistered and unregulated and do not follow a standard curriculum. A negligible proportion of them received twelve months training from a short-lived government sponsored programme (the 'Palli Chikitsok' (PC) training programme, which followed the China's model of barefoot doctors) in the '80s.

b. Drug store salespeople: most of have had no training in dispensing, not to speak of training in diagnosis and treatment.

3) Traditional healers: 'Kabiraj', whose practice is based on diet, herbs, and exercise. They are mostly self-trained, but some may have training from government or private colleges of Ayurvedic medicine. Some of them combine ayurvedic, unani (traditional muslim medicine originating from Greece) and allopathic medicine to provide 'totka' treatment. This category also includes non-secular faith healers.

4) Traditional birth attendants: includes both trained and non-trained providers who deliver home-based services only.

5) Homeopaths: mostly self-educated, but some possess a recognized qualification from government or private homeopathic colleges.

### The survey

The study passed the ethical review board of the James P. Grant School of Public Health, BRAC University for ethical approval. Informed consent was taken before interviewing. All enumerators hired for the survey underwent a five-day training which consisted of didactic lectures followed by practice sessions outside the study areas. The day-to-day field activities of the teams were overseen by a field researcher based in the Upazila (sub-district) field office. The whole survey activity was supervised and managed by the authors who made frequent field visits and provided assistance and guidance when needed. SPSS PC+ ver.12 was used for data analysis.

## Results

Table 2 presents the density (per 10 000 population) of doctors, nurses and dentists by region (division), geographical location (rural/urban) and sex (male/female). There were around five physicians and two nurses per 10 000 population, the ratio of nurse to physician being 0.4 only (i.e. 2.5 times more doctor than nurses). The ratio was equal in Khulna (1.4), but very low in Sylhet (0.1) and low in Dhaka (0.2). Substantial variation in the density of physicians and nurses among different divisions was found, Dhaka having the highest density of physicians followed by Chittagong, while in the case of nurses, this trend was reversed. Gross imbalance in density favouring urban areas was also observed, especially for the physicians. Similarly, there was also gross imbalance in sex ratio, favouring males in the case of physicians (four males to one female), and females in the case of nurses (nine females to one male). Together, there were 7.7 formally qualified registered health care professionals per 10 000 population.

**Table 2:** Distribution of doctors, nurses and dentists per 10 000 people in various Bangladeshi divisions

	Doctors	Nurses	Dentists	All	Nurse per Doctor ratio
Division					
Barisal	1.7	0.9	0.3	3.08	0.5
Chittagong	4.8	3.6	0.3	8.8	0.7
Dhaka	10.8	2.8	0.5	14.2	0.2
Khulna	1.3	1.9	0.05	3.3	1.4
Rajshahi	2.1	1.1	0.0	3.2	0.5
Sylhet	2.2	0.4	0.0	3.2	0.1
Location					
Rural	1.1	0.8	0.08	2.1	0.7
Urban	18.2	5.8	0.8	24.9	0.3
Sex					
Male	4.5	0.2	0.2	5.0	0.05
Female	0.8	1.8	0.03	2.7	2.1

All	5.4	2.1	0.3	7.7	0.4

The density of the other categories of allopathic health care providers (semi-qualified/unqualified) is presented in Table 3. There were around 12 village doctors and 11 sales people at drug retail outlets (providing diagnosis and treatment) per 10 000 population. Thus, there were about 2.5 times more village doctors and 2 times more drug store salespeople than were physicians who provide treatment/curative services to the population. There was not much variation in the density of the drug store salespeople between urban and rural areas (13 and 11 per 10 000 population) indicating their uniform spread across the country. However, their density was lowest in Barisal and Sylhet divisions compared to others. Also, there were twice as many CHWs from the NGO sector per 10 000 population (6) than from the government sector (3) and an overwhelming number of traditional birth attendants (TBAs) and/or trained traditional birth attendants (TTBAs). The TBAs/TTBAs were involved in providing delivery-related services at home only. The village doctors and the CHWs were mainly concentrated in the rural areas while the paraprofessionals were concentrated in the urban areas. Dhaka had the lowest number of village doctors and Sylhet the lowest number of CHWs than other divisions. The village doctors and the drugstore salespeople were predominantly male compared to the CHWs who were predominantly female.

**Table 3:** Distribution of semi-qualified and unqualified allopathic providers per 10 000 populations, various Bangladeshi divisions

	Semi-qualified allopathic providers*				Unqualified allopathic providers		
				
	Paraprofes-sionals	Community Health Workers					
		**Govt.**	**Non-Govt.(including traditional birth attendants)**	**All**	Village doctors** (rural medical practitioners/'Palli Chikitsok' village doctors)	**Dug store salespeople, drug vendors**	**All**

Division							
Barisal	0.7	4.5	37.9	42.4	15.5	6.5	22.1
Chittagong	1.6	4.6	50.9	55.5	17.1	8.7	25.8
Dhaka	0.8	2.6	26.6	29.2	9.8	11.9	21.8
Khulna	0.6	3.0	46.3	49.9	11.3	12.3	23.6
Rajshahi	1.3	3.0	48.3	51.4	13.5	13.4	26.9
Sylhet	0.6	3.8	41.4	45.3	12.7	6.3	19.0
Location							
Rural	0.8	3.6	49.5	14.1	13.8	10.8	24.6
Urban	1.6	2.0	10.1	12.1	8.8	13.2	22.1
Sex							
Male	0.3	1.2	0.2	1.4	12.0	11.0	23.0
Female	0.7	2.0	39.4	41.4	0.4	0.4	0.9

All	1.0	3.2	39.7	42.9	12.5	11.4	23.9

*received varying length of training from formal institutions, GO or NGO

**the Palli Chikitsok village doctors are included in this group because they are few in number, were trained on or before 1982 without any further re-training, and no different from the rural medical practitioners in practice

Finally, Table 4 presents the density of non-allopathic health care providers such as traditional healers and homeopaths. There were large numbers of faith healers as well as Kabiraj and other traditional healers (31 and 33 per 10 000 population respectively), who were providing health care services as revealed from this inventory. This was supplemented by 3 qualified and 2.5 unqualified homeopaths per 10 000 population in the country. The traditional practitioners were mostly male, concentrated in the rural areas of Chittagong, Rajshahi and Khulna divisions. On the other hand, the homeopaths were concentrated in the urban areas, mainly in the Khulna and Rajshahi divisions. Interestingly, about one provider (per 10,000 population) was engaged in delivering health related services such as circumcision, cleaning ears and extracting painful tooth at a nominal cost, mainly to the poorer section of the population.

**Table 4:** Distribution of traditional healers, homeopaths and others per 10,000 populations, various Bangladeshi divisions

	Traditional healers			Homeopaths			Others*
		
	Kabiraj, Totka	Faith healers	All	Qualified	Unqualified	All	
Division							
Barisal	12.8	17.7	30.5	1.0	2.3	3.3	1.7
Chittagong	49.3	40.6	89.9	3.0	1.6	4.7	2.2
Dhaka	29.6	20.3	49.8	3.6	1.2	4.7	2.6
Khulna	38.2	28.0	66.2	3.9	4.7	8.6	0.9
Rajshahi	35.7	45.8	81.6	3.6	3.9	7.5	1.1
Sylhet	14.9	38.1	53.1	3.8	2.2	6.1	0.0
Location							
Rural	42.1	40.5	82.6	2.5	2.9	5.5	1.05
Urban	4.4	4.2	8.6	6.1	0.9	7.0	4.02
Sex							
Male	23.4	22.2	45.6	3.2	2.3	5.5	1.4
Female	9.3	9.3	18.6	0.3	0.1	0.4	0.3

All	32.7	31.5	64.2	3.4	2.5	5.9	1.7

*Circumcision practitioners, ear cleaners, tooth extractors, etc.

## Discussion

Bangladesh is declared by WHO as one of the 58 crisis countries facing an acute HRH crisis [3]. However, this is given little importance in national health activities [ and there exists a dearth of information on these aspects at national level [14]. The Health Care Provider Survey 2007 [12] attempted to fill in this critical knowledge gap and help guide in formulating appropriate policies to improve the health system's ability to reach the people with an acceptable quality of services [15], and rational skill-mix in foreseeable future.

The survey is unique in that it had included all types of healthcare providers in the formal and informal sectors and thus presents a comprehensive picture of the healthcare scenario prevailing in the country. It used a nationally representative sample frame, and a PPS sampling strategy to take care of the size of the divisions and the rural/urban divides. However, due to constraint in time and resources, the number of sample clusters had to be limited to 60, a multiple of the six administrative divisions in the country.

### Shortage

Findings revealed that the density (per 10 000 population) of physicians and nurses has increased over the last decade (from 1.9 physicians and 1.1 nurses in 1998 to 5.4 physicians and 2.1 nurses in 2007) [9] though it remains much lower than the estimated average for low income countries in 1998 [16]. The density of dentists has also increased, but remains at a very low level (from 0.01 in 1998 to 0.3 in 2007). However, the density of formally qualified health care professionals (HCPs) (doctors, nurses and dentists) (7.7) is lower than other south Asian countries (e.g. 21.9 in Sri Lanka, 14.6 in India, and 12.5 in Pakistan) and falls far short of the estimate projected by WHO (23.0) which would be needed for achieving the MDG targets [3]. During this time, the density (per 10 000) of traditional birth attendants declined (from 55 in 1981 to 33 in 2007), presumably due to the stoppage of TBA training by the Government of Bangladesh in 1998 [17].

On the other hand, the increase in the number of unqualified allopathic providers during the past decade has been phenomenal compared to the qualified or semi-qualified allopathic providers. For example, the number of unqualified allopathic providers (village doctors and drug store salespeople) (24 per 10 000) has increased to about twice that estimated by the research agency, 'Org-Marg Quest' at the higher range (14.5 per 10 000) [18]. Similarly, the density of traditional healers (64 per 10 000) in this study has been found to be more than 2.5 times than the density estimated by Ali at the higher range (24 per 10 000) [19].

### Inappropriate skill-mix

The current nurse-doctor ratio of 0.4 (i.e. 2.5 times more doctors than nurses) is far short of the international standard of around three nurses per doctor. Interestingly, the equal nurse-doctor ratio in Khulna and very low nurse-doctor ratio in Sylhet is also associated with better health indicators in Khulna and worse health indicators in Sylhet. The importance of the nursing population for healthier communities (compared to individual outcomes in case of doctors) cannot be overemphasized [20]. There is also a gross imbalance in the doctor-technologist ratio as well, the ideal being five technologists for one doctor. An estimate of shortage based on the doctor-population ratio currently prevalent in low-income countries revealed a shortage of over 60 000 doctors, 280 000 nurses and 483 000 health technologists in Bangladesh [12].

### Inequitable distribution

It is interesting to note that the overwhelming urban bias of the distribution of the formally qualified HCPs, noted a decade ago, has remained a persistent phenomenon [16]. Also, these providers are inequitably concentrated in the Dhaka and Chittagong regions. The CHWs from the non-government sector and the village doctors are mainly concentrated in the rural areas. Interestingly, the salespeople at drug retail outlets (shops) are evenly distributed between the rural and urban areas, showing their unhindered expansion across the country. According to an estimate, there are about 80 000 unlicensed drugstores in the country [21]. This mushrooming of unregulated drug shops is facilitated by easy availability of essential drugs at low price following the National Drug Policy of 1982 [22] and also the availability of prescription drugs over-the-counter.

### Addressing shortage and skill-mix problems: what can be done?

The large-scale shortage of qualified healthcare providers, coupled with an inappropriate skill-mix (more doctors than nurses and technologists) needs urgent attention to cater to the healthcare needs of the population. While in the short-term it is nearly impossible to produce the huge numbers of estimated healthcare providers by the public and private sectors combined [12], the disease profile in the country does not always warrant provision of services by qualified health professionals. According to the Bangladesh Bureau of Statistics [23], the most common illnesses (both sexes) in order of frequency are: fever (55%), pain (10%), diarrhoea (6%) and dysentery (4%). The above pattern of disease burden, at least in the primary care level, can be handled by the paraprofessionals (medical assistants, family welfare visitors (FWVs)), including CHWs, with the establishment of a functional referral system to a higher level of facilities [24,25].

The CHWs have been increasing in size since the nineties, with the expansion of the government and NGO health network in the country. They have been found to be cost-effective [26,27] and useful in the management of childhood pneumonia [28], acute respiratory infections of children [29], screening childhood hearing impairment [30], and DOTS treatment of tuberculosis [31] in rural Bangladesh. Training may also be provided to improve the competency of the vast army of unqualified providers (especially village doctors) in rational and harmless healthcare provision [32]. Any concern that upgrading their diagnostic and curative skills may lead to abuse and malpractice may be contained by managerial and regulatory interventions by the public sector [33].

## Conclusions

Bangladesh is suffering from a severe HRH crisis in terms of a shortage of qualified providers (when measured against the WHO estimate for achieving MDG targets), inappropriate skills-mix and inequity in distribution. This desperate situation demands immediate attention from policy makers. Reducing the 'income-erosion' effect of illness through a pro-poor health system is urgently needed in Bangladesh, a country besieged with large out-of-pocket payments for healthcare.

## Competing interests

We declare that we have no competing interests in conducting the research and writing the manuscript.

## Authors' contributions

SMA, AMR, and AB conceptualized and designed the study; MAH helped in sampling and fielding the study. SMA and MAH analysed and interpreted the data; AMR and AB also helped in its interpretation. SMA drafted the manuscript and MAH, AMR and AB put critical inputs in improving the draft. SMA revised and prepared the final draft. All authors read the final draft and approved it for submission.
